# Evaluation of Patterns of Recurrences in Patients With Breast Carcinoma: Are We Treating the Right Volumes, or Does the Tumor Biology Play the Role?

**DOI:** 10.7759/cureus.102214

**Published:** 2026-01-24

**Authors:** Prabha Verma, Rohini Khurana, Siddharth Kumar, Avinash Poojari, Madhup Rastogi, Niraj Agarwal, Ajeet K Gandhi, Avinav Bharati, Kailash Mittal, Smriti Srivastava

**Affiliations:** 1 Radiation Oncology, Uttar Pradesh (UP) University of Medical Sciences, Saifai, IND; 2 Radiation Oncology, Dr. Ram Manohar Lohia Institute of Medical Sciences (Dr. RMLIMS), Lucknow, IND; 3 Radiation Oncology, Viswabharathi General Hospital and Medical College, Kurnool, IND; 4 Medical Oncology, Max Super Speciality Hospital, Lucknow, IND; 5 Radiation Oncology, Dr. Ram Manohar Lohia Institute of Medical Sciences (DR. RMLIMS), Lucknow, IND; 6 Radiation Oncology, All India Institute of Medical Sciences, Delhi, IND; 7 Radiology/Radiation Oncology, Uttar Pradesh (UP) University of Medical Sciences, Saifai, IND; 8 Radiation Oncology, Hind Institute of Medical Sciences, Lucknow, IND

**Keywords:** 3d conformal radiation therapy, breast cancer, cancer therapy and biology, radiation therapy oncology group (rtog), regional nodal irradiation, tumor recurrence

## Abstract

Purpose

The study's purpose is to evaluate the patterns of locoregional recurrences (LRRs) in women with breast cancer treated with curative intent surgery with conventional postoperative radiation therapy and to assess whether LRRs are mainly related to clinical target volume (CTV) coverage, tumor biology, or both.

Materials and methods

This was a retrospective observational study conducted between 2016 and 2023; 151 patients with histopathologically proven infiltrating ductal carcinoma (IDC) of the breast were included in the study. After modified radical mastectomy or breast-conserving surgery (BCS) with axillary dissection or sentinel lymph node biopsy, based on the indications, patients also received regional nodal irradiation (RNI). Patients were treated with 50 Gy in 25 fractions with an additional 10-16 Gy boost in 5-8 fractions in BCS patients by conventional field border-based plans on the linear accelerator. Patients who developed LRRs were studied and mapped for patterns of recurrences and compared with the Radiation Therapy Oncology Group (RTOG) guidelines of CTV delineation. All cases of bilateral breast cancer were excluded. In all cases, regional RNI, including supraclavicular and axillary lymph node irradiation, was done, while internal mammary chain (IMN) irradiation was done in 19% of cases only.

Results

Median follow-up was 60 months. Of 151 patients, 15 (10%) developed LRRs. When compared to the index population, 40% of the patients in the recurrence group had triple-negative breast carcinoma versus 35.1% in the index population group, and 27% in the recurrence group were human epidermal growth factor receptor 2 (HER2)-positive versus 17% in the index group. Five-year LRR-free survival, distant metastasis-free survival, and overall survival were 90.07%, 82.79%, and 89.41%, respectively. Most of the patients with recurrences had aggressive biological features with IDC grade 3 tumors in 10/15 (67%), >4 node-positive disease in 15/15 (100%), triple-negative tumors in 6/15 (40%), and HER2neu 3+ disease in 4/15 (26.67%) (three out of the four patients had taken one year of anti-HER2 therapy also). Lymphovascular invasion was observed in 10/15 cases (67%). In 10 (67%) cases, LRRs were diagnosed simultaneously as the metastatic disease, while five (33%) patients presented with distant metastases secondarily. Chest wall (local) recurrences occurred in 12 (80%) cases, which also had a marginal failure, i.e., at the posterior border of RTOG volumes; 13 (87%) regional recurrences were observed in 11 patients; of these, seven (53.8%) recurrences occurred in the supraclavicular fossa. Four (31%) recurrences occurred inside the RTOG level III axilla, and two (15%) recurrences occurred inside the RTOG volume in the IMN. Of all 13 regional recurrences, only 3/13 (23%) regional recurrences occurred outside RTOG CTV, while 10 (77%) recurrences occurred inside RTOG volumes.

Conclusion

Our study showed that LRRs predominantly occurred in patients with aggressive tumor biology. Approximately 70% of failures were covered inside RTOG volumes, which indicates that if RTOG volume-directed planning had been used for radiation treatment in such high-risk patients, these geographical misses could have been avoided. Adopting RTOG guidelines for volume delineation in high-risk cases with aggressive histology might be beneficial. However, further follow-up and meticulous documentation of the recurrences are needed to improve their understanding.

## Introduction

Breast cancer is the most common cancer diagnosed among women worldwide and in India and the leading cause of cancer death, with an estimated 2.3 million new cases representing 11.5% of all cancer cases in 2022. Among women, breast cancer accounts for one in four cancer cases and one in six cancer deaths [1]. Breast cancer involves multimodality management, including surgery, chemotherapy, hormonal therapy, targeted therapy, and radiation therapy (RT). Breast/chest wall (CW) radiotherapy forms an important component of adjuvant treatment in the management of patients with breast cancer. Evidence from several randomized studies supports the use of whole breast radiotherapy (WBRT) with a boost in breast conservation therapy (BCT) and post-mastectomy radiotherapy (PMRT) for women with high-risk breast cancer [2,3]. However, there have been controversies in treating regional lymph nodes (LN). Earlier studies suggested that women with ≥4 involved lymph nodes should receive regional nodal irradiation (RNI targeting the internal mammary chain {IMN}, axillary, and supraclavicular fossa {SCF} nodal regions). However, the long-term follow-up of these trials has shown a locoregional control (LRC) and survival benefit even in patients with 1-3 involved lymph nodes [4-6]. Several randomized trials (National Cancer Institute of Canada {NCIC} MA.20 trial, DBCG-IMN study, and EORTC 22922/10925 trial) have also enumerated an improvement in locoregional disease-free survival (DFS), distant DFS, and overall DFS after regional nodal irradiation (RNI) in all node-positive patients with breast cancer or node-negative patients with pathological high-risk features [7-9].

Although conventional radiation treatment fields for PMRT, used in the aforementioned studies, provide excellent oncological outcomes, they can result in heterogeneous dose distribution as bony landmarks used for field boundaries often have little anatomical relation with the draining lymphatics [10]. Moreover, it is assumed that tangential fields cannot ensure lower axillary optimal nodal coverage because of different patient anatomy; therefore, the contouring of regional nodes is necessary if the lower axilla is intended to be included in radiation treatment, especially in cases of patients with heavy nodal burden breast cancer [11]. Nowadays, 3D computed tomography (CT)-based planning for patients with breast cancer is evolving, and with the advancement of more conformal techniques such as 3D conformal radiation therapy (CRT) and intensity-modulated radiation therapy, an evident need exists to optimize the existing delineation guidelines. There are various guidelines available for clinical target volume (CTV) delineation, such as the radiotherapy oncology guidelines (Radiation Therapy Oncology Group {RTOG}), European Society for Radiotherapy and Oncology (ESTRO) guidelines, Danish guidelines, and PROCAB guidelines [12-15].

These delineation guidelines incorporate soft tissue/vascular anatomy-based delineation for breast/chest wall (CW) and individual regional lymph node stations (axillary, SCF, and IMN) and are more conformal to draining lymphatics [12-15]. Out of the available guidelines, RTOG guidelines use soft tissue/muscular landmark-based delineation and are the most widely accepted and used. Various studies suggest that using RTOG guidelines for target delineation may improve dose coverage to regional lymph nodes, breast/CW, with no significant increase in doses to organs at risk (OARs) [16-18]. So, we used RTOG guidelines to evaluate the pattern of regional recurrences.

The primary objective of the present study was to evaluate locoregional recurrences and to describe the patterns of locoregional failure in women with breast cancer irradiated by a previously described post-mastectomy conventional radiation technique. The secondary objective was to analyze the tumor characteristics and radiation volumes/doses that may have resulted in locoregional failure. The essential question investigated in this study is whether locoregional recurrence is mainly related to clinical target volume (CTV) coverage or to tumor biology.

The abstract of this article was previously presented as a meeting abstract at the ESTRO 2025 Annual Scientific Meeting held in Vienna, Austria, from 2 to 6 May 2025.

## Materials and methods

This retrospective observational study analyzed data from 151 female patients aged 18-80 years with histopathologically confirmed infiltrating ductal carcinoma (IDC) of the breast, treated at a tertiary care center between July 2016 and June 2023. Patients with no distant metastasis (DM) at presentation who underwent either mastectomy or breast-conserving surgery (BCS) and subsequently received postoperative radiotherapy were included. The median follow-up duration was 60 months.

All cases of bilateral breast cancer, patients with distant metastasis before the start of radiation, patients requiring electron beam therapy, patients with a history of non-cutaneous malignancies, patients with incomplete medical records, patients who were lost to follow-up for more than six months or patients who can not be contacted, patients with contralateral nodal recurrence (NR) or chest wall or breast-only recurrence without nodal recurrence, patients undergoing hypofractionated RT, and male patients with breast cancer were excluded from the study.

All the patients underwent either modified radical mastectomy with axillary lymph node dissection (ALND) or sentinel lymph node biopsy (SLNB) or breast-conserving surgery (BCS) with axillary lymph node dissection or SLNB. Chemotherapy was administered to all the patients in the neoadjuvant or adjuvant setting. In cases with the presence of human epidermal growth factor receptor 2 (HER2) overexpression, combination therapy with trastuzumab is also used along with chemotherapy. Hormone therapy was given to all the patients with hormone receptor-positive disease.

Postoperative radiation therapy was given in cases with T3-T4 disease and/or any T with 1-3 lymph node-positive disease and in cases of BCS and/or in the presence of a combination of two risk factors (age of <40 years, multifocal disease, lymphovascular invasion (LVI), and grade 3, HER2+, triple-negative, and pT2 tumors). Based on the indications, regional nodal irradiation (RNI) was also done, including the supraclavicular fossa (SCF) and axilla regions (levels I, II, and III), and in around 19% of cases, internal mammary chain (IMN) irradiation was also done.

All the patients were treated with conventional field border-based plans using parallel opposed tangents with matched SCF field, using a mono-isocentric technique, using multileaf collimators (MLCs) on the linear accelerator (Linac) machine. Doses of 50 Gy/25 fractions at the rate of 2 Gy/fraction for five fractions per week were delivered to the chest wall and regional lymph nodes, respectively, with an additional 10-16 Gy/5-8 fraction boost in cases of BCS patients by conventional field border-based plans on the Elekta Synergy Linac machine. Wherever required, a personalized bolus was designed according to the thickness of the chest wall.

The primary aim of our study was to conduct a comprehensive mapping analysis of locoregional recurrences observed in 15 (10%) patients. Correlative imaging available at the time of recurrence provided detailed spatial information critical for this analysis. In addition to mapping NR locations, we also tried to validate the clinical target volume (CTV) contouring guidelines as recommended by the RTOG consensus documents. Using our robust dataset, we assessed how well these RTOG guidelines covered the regions at risk for recurrence and the association of clinical and pathological factors with the pattern of recurrences.

The axilla was treated only in cases with more than 50% of positive lymph nodes on the resection specimen, with extranodal extension positive, when lymph node dissection was not performed, or with inadequate lymph node dissection with node-positive disease. Supraclavicular fossa (SCF) irradiation was done for all the node-positive cases. IMN irradiation was done in cases with an inner quadrant or centrally located tumor in the presence of any two of the following four criteria: age of ≤40 years, tumor size of >2 cm, LVI, and in cases with more than 50% of positive lymph nodes on the resection specimen. Wide tangential beams were used wherever IMN irradiated.

All these 151 patients were referred to as the index group in the study. For each patient, radiation treatment data regarding patient positioning (from photos during simulation CT), previous treatment CT images, radiation fields, dosimetry, organ-at-risk (OAR) shielding, and bolus characteristics were retrieved from technical files.

In cases that developed local or regional recurrences during follow-up, patterns of recurrences were mapped. All these 15 patients with recurrences were referred to as the recurrence group in the study. Diagnostic CT scans or positron emission tomography (PET)-CT scan data were collected in a standard simulation positioning, wherever possible, for patients with locoregional recurrences. The second step consisted of determining the precise site of node metastases based on computed tomography (CT) and positron emission tomography (PET) data. In all the patients, radiographic features of malignancy, such as those nodes with a short axis diameter of >1 cm, the extent of fluorodeoxyglucose-avidity (if PET was used), infiltrative borders, and heterogeneous enhancement with central necrosis, were used as criteria to determine a nodal recurrence (NR). All NRs were then overlaid onto pre-treatment CTs (image fusion), and RTOG guideline-based CTV contours were retrospectively overlaid on baseline pre-treatment CTs. Then, based on the consensus of a group of reviewers (to reduce the chances of inter-observer variability), geographical spatial sites of regional recurrences were defined according to RTOG guidelines of CTV delineation [5,12].

Data from all patients were analyzed for patient and tumor characteristics, treatments received, treatment duration, and survival. Overall survival (OS) was defined as the time between the end of radiation therapy and the date of last follow-up. Locoregional recurrence-free survival (RFS) and distant metastasis-free survival (DMFS) were defined as the time from the end of radiotherapy treatment until locoregional recurrence or the date of metastases, respectively. In all 151 patients, multivariable analysis was performed to identify clinical and pathological features associated with nodal recurrences. The Kaplan-Meier analysis method was used for estimating survival data. All the statistical data were analyzed using IBM SPSS statistical software version 20.0 (IBM Corp., Armonk, NY).

## Results

At a median follow-up of 60 months, 15 (10%) out of 151 patients developed locoregional recurrences. The median age of the patients who developed recurrences (recurrence group) was 53 years, and around 67% were post-menopausal. Around 67% of the patients were post-mastectomy, and 33% of the patients were post-breast-conserving surgery. About 40% of the patients in the recurrence group received neoadjuvant chemotherapy (NACT) before surgery, and 60% underwent surgery afferently. All the patients with recurrences were stage IIIA and above. When compared to the index population (151 patients), 40% of the patients in the recurrence group had triple-negative breast carcinoma (TNBC) versus 35.1% in the index population group, and 27% were human epidermal growth factor receptor 2 (HER2)-positive in the recurrence group versus 17% in the index group. Patient characteristics are enumerated in Table 1.

**Table 1:** Characteristics of all patients (index population) in the registry (first line) and those identified to have locoregional recurrences as a site of first failure (second line) BCS, breast-conserving surgery; MRM, modified radical mastectomy; TNBC, triple-negative breast carcinoma; ER, estrogen receptor; PR, progesterone receptor; HER2neu, human epidermal growth factor receptor 2/neu proteins; IDC, infiltrating ductal carcinoma; NACT, neoadjuvant chemotherapy; f/b, followed by

Patient characteristics		All cases (index group)		Locoregional recurrent cases (recurrence group)	
		Number=151	Percentage (%)	Number (15)	Percentage (%)
Median age	48 years (32-70)			53 years	
Laterality	Left	86	57%	10	67%
	Right	65	43%	5	33%
Surgery	BCS	47	31%	5	33%
	MRM	104	69%	10	67%
Quadrant	Outer	115	76%	10	67%
	Inner	36	24%	5	33%
Stage (index)	IIA	15	15%	0	0
	IIB	35	22%	0	0
	IIIA	62	40%	9	60%
	IIIB	29	16%	3	20%
	IIIC	10	7%	3	20%
IDC grade	1	7	5%	0	0
	2	69	45%	5	33%
	3	75	50%	10	67%
Lymph node	<4	101	67%	6	40%
	≥4	50	33%	9	60%
Timing of surgery	NACT f/b surgery	73	48.3%	6	40%
	Upfront surgery	78	52%	9	60%
Menopausal status	Post-menopausal	80	53%	10	67%
	Pre-menopausal	71	47%	5	33%
Immunohistochemistry (IHC) status	TNBC	53	35.1%	6	40%
	ER+/PR+/Her2neu	48	32%	1	7%
	ER-/PR-/Her2neu 3+	26	17%	4	27%
	ER+/PR-	8	5%	2	13%
	Triple+	16	11%	2	13%
Median radiation dose	50.0 Gy (40-66 Gy)			50.0 Gy (40-52.5 Gy)	
Median radiation time	BCS 42 days			BCS 42 days	
	MRM 35 days			MRM 35 days	
Median follow-up	60 months (15-96)			44 months (16-96)	

Five-year locoregional recurrence-free survival, distant metastasis-free survival, and OS were 90.07%, 82.79%, and 89.41%, respectively. Most of the patients with recurrences had aggressive biological features with IDC grade 3 tumors in 10/15 patients (67%), >4 node-positive disease in 9/15 patients (60%), and triple-negative tumors in 6/15 (40%) patients, while 4/15 patients, i.e., (26.67%), were HER2neu 3+, and the remaining were hormone receptor-positive. LVI was observed in 10/15 cases, i.e., 67% of patients with recurrences. In 10 (67%) cases, the locoregional recurrences were diagnosed simultaneously as the metastatic disease, whereas five (33%) patients presented with distant metastases secondarily.

It is striking that nine out of 15 patients, i.e., 60% of our study cohort, exhibited ≥4 node-positive disease at presentation. All the patients with recurrences were stage IIIA and above, around 10 (67%) patients had IDC grade 3, and six out of 15, i.e., 40% of the patients, had TNBC, suggesting that factors in addition to nodal status are important in determining recurrence risk. We attempted to identify which clinical and pathological characteristics are associated with NRs by region, as such findings would enable the personalization of contouring regional nodes for breast cancer radiotherapy. We found that estrogen receptor (ER)/progesterone receptor (PR)-negative status, LVI, grade 3 disease, and ≥4 node-positive disease independently predicted SCF and IMN recurrence. Positive lymph nodes in the adjacent axilla were also associated with posterior SCF NR. No clinicopathologic features were identified that specifically differentiated the patients who would recur in the posterior SCF region relative to those elsewhere in the SCF.

Patterns of locoregional recurrences

**Figure 1:**
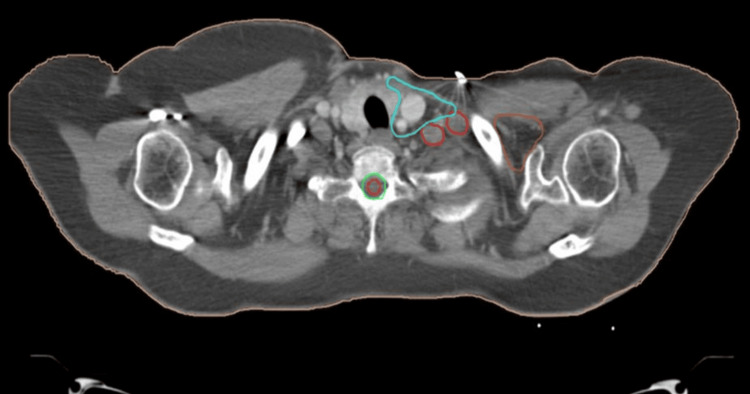
CT of the thorax axial image showing SCF recurrence as nodular deposits (denoted by red color) at the posterolateral border of RTOG SCF CTV (cyan color), showing the most common site of recurrence in the SCF region, which needs to be carefully covered during radiation planning CT, computed tomography; SCF, supraclavicular fossa; RTOG, Radiation Therapy Oncology Group; CTV, clinical target volume

**Figure 2:**
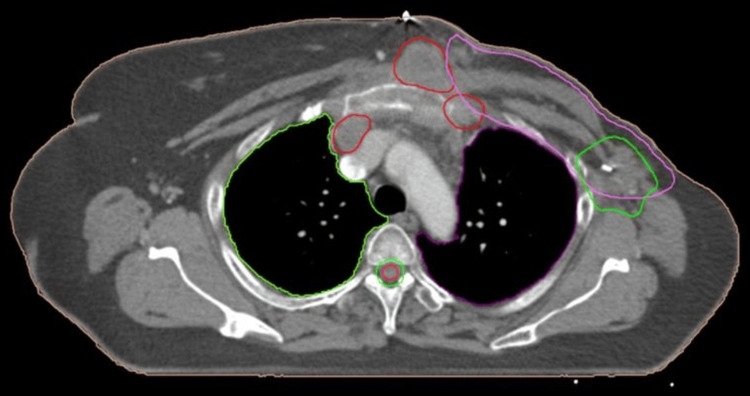
CT of the thorax axial image showing chest wall soft tissue deposits on the left side (red color) at posterior-medial margins and at the chest wall-rib interface of RTOG chest wall CTV (purple color) denoting marginal failure in the given case; another soft tissue deposit (red color) is also seen in the anterior mediastinum (right side), suggestive of distant metastasis CT, computed tomography; RTOG, Radiation Therapy Oncology Group; CTV, clinical target volume

Chest wall recurrences occurred in 12 out of 15 patients with recurrences, i.e., in 80% cases. When we mapped these recurrences with respect to RTOG contours, all these recurrences were seen at the posterior border of RTOG volumes. Thirteen (87%) regional recurrences were observed in 11 patients; out of these, seven (53.8%) recurrences occurred in the SCF region (three {42.8%} SCF recurrences were outside RTOG CTV, and four {57.2%} SCF recurrences occurred at the posterolateral border of SCF), and four (31%) recurrences occurred in level III axilla, but when seen with relation to RTOG volumes drawn, it was found that these recurrences were coming inside RTOG volumes, and two (15%) recurrences were seen in the IMN region, but these two recurrences were also coming inside RTOG contours (Figures 1, 2).

Only 3/13 (23%) regional recurrences occurred outside the RTOG contoured volume, while 10 (77%) recurrences occurred inside/at the margins of the RTOG volumes. Previous treatment plans were also retrieved to detect areas of recurrence with respect to 90% isodose line of conventional treatment plans and found that around 77% of these recurrences were at the edges of 90% isodose line, and around 33% recurrences were outside of 90% isodose line of conventional plans, suggesting that even when high-risk nodal regions are clinically targeted, biological aggressiveness may override the benefits of anatomical coverage.

## Discussion

Several randomized trials have demonstrated survival benefits of RNI in women with node-positive breast cancer, justifying its selective use in this population. Data from these trials have also demonstrated an increase in locoregional control rates and overall survival among women with 1-3 node-positive breast cancer [6,19]. With the advancements in radiation therapy and highly conformal radiation techniques, there is an increasing need for the accurate delineation of nodal regions. However, one drawback with these modern techniques is that they exhibit steep dose gradients, often resulting in the underdosing of the regions that previously received therapeutic doses with 2D and 3D conformal radiation techniques. Consequently, precise target volume delineation has become critical to ensure oncological safety.

For a very long time, risk-based axillary management has been a critical focus in breast cancer treatment. In the NSABP B-04 trial, axillary radiation therapy (RT) proved to be as effective as axillary lymph node dissection (ALND) for patients with clinically node-negative disease who did not receive systemic therapy [20]. The After Mapping of the Axilla: Radiotherapy or Surgery? (AMAROS) trial, a phase 3 randomized, multicenter, open-label trial, also compared the effectiveness of axillary lymph node dissection (ALND) with axillary radiotherapy (ART) for patients with early-stage breast cancer and a tumor-positive sentinel lymph node, and the study found that ART provides comparable axillary control with significantly less lymphedema (arm swelling), suggesting that ART is a preferred option for reduced morbidity in selected patients [21].

Practical review from the Transatlantic Radiation Oncology Network (TRONE) for the evaluation of locoregional relapse patterns by comparing various contouring guidelines suggested that the RTOG atlas may more adequately cover the areas at risk for patients with locally advanced disease or patients with high-risk features such as T3/T4 tumors, extracapsular extension, LVI, multiple positive nodes, and triple-negative disease [22]. However, as the RTOG target volumes are larger, it is assumed that using RTOG guidelines for radiation planning may lead to an increase in doses to the surrounding organs at risk (OARs). However, several studies have indicated that using RTOG guidelines for volume delineation may lead to improved coverage in high-risk areas compared to conventional radiation techniques with a nonsignificant increase in dose to OARs [18]. Moreover, these are most commonly used guidelines, so we used RTOG guidelines for volume delineation and for mapping the patterns of recurrences.

At a median follow-up of 60 months, 15 (10%) locoregional recurrences were seen; of these, 12 (80%) recurrences were chest wall recurrences, and 13 out of 15, i.e., 87% recurrences, were regional recurrences. It was seen that SCF was the most frequent site of regional recurrence, accounting for 53.8% recurrences, which aligns with the regional failure patterns seen in many clinical trials.

Conventional SCF fields are mainly field border-based and extend inferiorly to the acromial-clavicular joint, medially to the pedicle of the vertebral bodies, and laterally to the femoral head. A study by Jabbari et al. stated that the lateral and posterior borders of SCF are variably covered with conventional SCF fields and are dependent on where the lateral-most edge of the SCF field is placed and the depth to which the SCF prescription dose is prescribed, respectively [23].

Currently available contouring guidelines successfully cover the majority of NRs identified in our study. However, there are discrepancies in taking the superior border of SCF between different guidelines, as ESTRO guidelines suggest that only the lowest part of the SCF region should be considered as part of the CTV in elective LN irradiation in breast cancer [14]. In a study by Chang et al, with the use of ESTRO guidelines for volume delineation, high rates of geographical misses (28.8%) were seen in advanced-stage patients, so it was suggested that the expansion of the CTV is warranted for patients with locally advanced disease [22]. So, it was concluded that ESTRO guidelines might be helpful in patients with early-stage, low-risk breast cancer but are not intended for cases with locally advanced disease [22,24].

In our study, 13 (87%) regional recurrences were observed in 11 patients; out of these, seven recurrences (i.e., 53.8%) of all regional recurrences occurred in the SCF region. Four (57%) SCF recurrences occurred at the posterolateral border of SCF, and three SCF recurrences, i.e., 42.8% recurrences in the SCF region, were outside RTOG CTV. In a Korean mapping study of 162 NR among 234 patients, the SCF was the most common site of regional recurrence; however, in that study, about 20% of SCF recurrences were located posterolateral to the anterior scalene muscles, i.e., outside the RTOG CTV definitions, and medial SCF (level IV) accounted for about 33.8% of all regional recurrences, which was a bit contradictory to our study, as the posterolateral SCF region was the most common site of metastasis in our study; this discrepancy in results could be due to the small number of patients in our study [22].

In another series from MD Anderson Cancer Center, 21% of SCF NRs were located in the posterior or lateral SCF, which again supports our results that posterolateral SCF is the most common site of SCF recurrence [25]. A study from China described that a total of 50 (91%) and 45 (81.8%) patients had lymph node metastasis in the medial and lateral SCF subregions, respectively, and stated that lateral and posterior borders were the most overlooked locations in patients with breast cancer. In that study, even the RTOG atlas could cover only 62.6% of all nodal recurrences in the SCF region. This study suggested that the modification of the posterolateral SCF border by extending the borders to natural anatomical barriers in high-risk or recurrent patients might be a reasonable approach for increasing coverage [26].

In a study by Brown et al. of the 161 nodal metastases in the SCF region, 95 (59%) were within the RTOG consensus volume, four nodal metastases (2%) in three patients were marginally within the volume, and only 62 nodal metastases (39%) in 30 patients were outside the volume. Outside the RTOG SCF, recurrences occurred in the posterolateral SCF and in the lateral low SCF, and in women with multiple supraclavicular metastases, recurrences were also seen at the level of the cricoid and thyroid cartilage (superior to the RTOG volume). This study also supported that posterolateral SCF is the most common site of SCF recurrence in patients with breast cancer. Modifying the CTV to encompass the lateral and posterior SCF in patients with high-risk features might be beneficial to prevent recurrences [27].

In our study, around four (31%) recurrences occurred inside the RTOG level III axilla; all four of these patients had >4 node-positive disease, suggesting that regional recurrences in the axilla might be attributed to axillary nodal burden. In a similar study by Brown et al., it was seen that 92% of the lymph nodes in the posterior triangle in that series were seen in patients who had multiple supraclavicular nodal metastases, which means that regional recurrence in the axillary apex and SCF is associated with axillary nodal burden [27]. In a study by Strom et al., 1031 patients treated with mastectomy and chemotherapy were followed; out of these, 21 patients recurred within the low-mid axilla, and around 77 patients had recurrence in the SCF/axillary apex. The above study suggested that patients with ≥4 involved axillary lymph nodes, >20% involved axillary nodes, or gross extranodal extension are at an increased risk of failure in the SCF/axillary apex and should receive radiation to undissected regions in addition to the chest wall [28].

We observed two (15%) IMN region recurrences in our patients, which were also inside RTOG volumes. That suggested that if RTOG target volumes were used for target volume delineation in the above patients, probably IMN region recurrences would have been prevented in our patients. Consistent with other reports, the majority of IMN NRs were located within the first 1-3 intercostal spaces, with no IMN NRs occurring caudal to the fourth rib [7,23]. Unlike other studies that have examined patterns of IMN NRs, we did not find the association between IMN NR events and the quadrant of the primary lesion [29]. However, we did observe IMN NRs abutting the sternum, around 5 mm medial to the internal mammary vessels, suggesting that CTV coverage must extend all the way to the sternal border or at least up to the third intercostal space in cases of IMN irradiation.

Out of all 13 regional recurrences, only three (23%) regional recurrences were seen outside RTOG CTV, while 10 (77%) regional recurrences occurred inside RTOG volumes and at the edges of the 90% isodose lines in conventional plans. Since, in the study, RTOG contours were not used during treatment planning initially, it might be inaccurate to label intra-RTOG recurrences as "not missed." Rather, they reflect failures despite intended coverage, likely due to biological resistance, microscopic spread beyond planned fields, or dose fall off at junctions or probably due to small treatment volumes.

The maximum recurrences that were seen had stage IIIA and above, high-grade, heavy nodal burden, i.e., ≥4 node-positive disease and TNBC/Her2neu 3+ disease, depicting poor prognosis in these patients. This theory of biological aggressiveness is also supported by a similar study by Loganadane et al., where 796 patients with breast cancer were initially treated with post-mastectomy conformal electron beam therapy. When these patients were evaluated for patterns of recurrences, it was found that local recurrences were related mostly to the tumor's biological aggressivity and radioresistance, and only a small number of recurrences were caused by geographical miss [30].

The development of an NR and distant metastasis (DM) is often inextricably linked, making it difficult to discern which event comes first [24]. In our study, 10 out of 15, i.e., 67% of the patients, developed DM concurrently (within one month of the diagnosis of NR). Furthermore, in the recurrence group, among patients with lateral or posterior SCF nodal recurrence (NR), five (71%) patients developed DM concurrently, suggesting that SCF NR in these locations might be markers of other regional nodal metastasis and distant metastasis. But larger-scale, independent datasets are required to confirm this hypothesis. Details of the literature review are given in Table 2.

**Table 2:** Comparison of the present study with previous literature ESTRO, European Society of Radiation Oncology; RTOG, Radiation Therapy Oncology Group; NA, not available; TNBC, triple-negative breast carcinoma; HER2, human epidermal growth factor receptor 2; IDC, infiltrating ductal carcinoma; IMN, internal mammary chain; SCF, supraclavicular fossa; CT, computed tomography; Linac, linear accelerator

Literature review			
Study	Loganadane et al. [30] (retrospective series). *Clinical and Translational Radiation Oncology* (2017). doi:10.1016/j.ctro.2017.03.006	DeSelm et al.[31] (retrospective trial). International Journal of Radiation Oncology - Biology - Physics (2018). doi:10.1016/j.ijrobp.2018.10.021	Current study (retrospective study)
Objective	To evaluate locoregional control and patterns of locoregional failure in women with breast cancer irradiated by post-mastectomy electron beam radiotherapy (PMERT)	To map the anatomical pattern of isolated nodal recurrences (NR). To assess clinical and pathological factors associated with patterns of NR and survival rates	To evaluate the patterns of locoregional recurrences in women with breast cancer treated with radiation therapy on the Linac machine. To assess whether locoregional recurrences are mainly related to clinical target volume (CTV) coverage or to tumor biology or to both
Content	Patterns of locoregional recurrences compared to the ESTRO and RTOG guidelines of volume delineation	Mapping patterns of only regional recurrences were studied and compared to the ESTRO and RTOG guidelines of volume delineation	Patterns of locoregional recurrences were studied and compared to the RTOG guidelines of volume delineation
Patients in the study	796 women included. All women irradiated by PMERT	13042 eligible patients analyzed in the study	151 patients included, all women treated with conventional field border-based plans on Linac
Median follow-up	Median follow-up was 64 months	NA	Median follow-up was 60 months
Recurrences	23 patients (2.9%) presented with locoregional recurrences	153 eligible patients were mapped for locoregional recurrences	15 (10%) patients developed locoregional recurrences
Histopathological factors associated with recurrence	Patient's stage not mentioned/specified, 52% of the patients had TNBC in the recurrence group, <1% were HER2-positive, ≥4 positive lymph nodes in 43% patients, IDC grade 3 tumors in 74% of the patients, and lymphovascular invasion (LVI) observed in 48% cases	Stage not mentioned/specified; 29% of the patients had TNBC, 14% were HER2-positive, ≥4 positive lymph nodes in 17% of the patients, IDC grade 3 tumors in 68% of the patients, and lymphovascular invasion observed in 51% cases	All patients were stage IIIA and above, 40% of the patients had TNBC, 27% were HER2-positive, ≥4 positive lymph nodes in 100% of the patients, IDC grade 3 tumors in 67% of the patients, and LVI observed in 67% of the cases
Locoregional recurrence pattern	Chest wall (local) recurrences occurred in (56%) cases and regional recurrences seen in 65% of the patients with recurrences	Chest wall recurrences not mapped, regional recurrences seen in all patients, 25.5% recurrence in the SCF region, 42% axillary recurrence, and 32.5% IMN recurrence in the patients	Chest wall (local) recurrences occurred in (80%) cases, regional recurrences were seen in 87% of the patients with recurrences, 53.8% recurrences occurred in the SCF region, 31% recurrences occurred in level III axilla, and 15% recurrences occurred in the IMN region
Drawbacks	Retrospective nature. Small population of patients with local or regional recurrence. Patients treated with electron beam therapy and the coverage of ESTRO and RTOG volumes were compared retrospectively on CT images of patients with recurrences. Methodology not clearly described	Retrospective study. All cases of NR were mapped manually using a template CT. It is unclear how many patients received RT. As per the study, some patients did not receive postoperative RT. Recurrences studied on a representative template CT, which may or may not correlate with the patient's anatomy	Retrospective study. limited patient number. All patients treated with conventional field border-based radiation plans on the Linac machine. Recurrences studied on pre-treatment CTs after image fusion. Recurrence areas better defined and more correlative to the patient's anatomy

Limitations of the study

The findings of our study, as well as those of the aforementioned studies, should be interpreted in light of several inherent limitations. First, the retrospective identification of cohorts renders the analysis susceptible to selection and sampling bias. Notably, the observed locoregional recurrence rate of approximately 10% is higher than that reported in contemporary literature, which may reflect selection bias at the time of sample inclusion. Additionally, this was a retrospective, single-arm study designed to evaluate recurrence patterns in previously treated patients; consequently, variability in follow-up schedules and methods of recurrence detection may have contributed to inaccuracies in the reporting of nodal recurrences.

Furthermore, all patients were treated using conventional field border-based radiation planning on a linear accelerator. RTOG target volumes were contoured retrospectively solely to assess whether recurrence sites fell within these standardized volumes. So, recurrences coming inside the RTOG volumes might not be truly representative of in-field recurrences; rather, inside RTOG volume recurrences suggest that if the RTOG target volume might have been used for planning purposes, these recurrences might have been prevented. A prospective study with RTOG target volume-directed treatment plans would have given a clearer picture of patterns of recurrences.

## Conclusions

The current study denoted that high-risk patients who received regional RT relapsed at a higher rate compared to low-risk patients who received regional RT. So, it remains possible that women with stage IIIA and above, high-grade, heavy nodal burden, i.e., ≥4 node-positive disease and TNBC/Her2neu 3+ disease, may require different clinical target volumes (CTVs) compared to those receiving elective RT for recurrence prevention. Our study showed that locoregional recurrences predominantly occurred in patients with aggressive tumor biology, and many arose inside RTOG volumes, but at the edges of conventionally treated fields, suggesting that even when high-risk nodal regions are clinically targeted, biological aggressiveness may override the benefits of anatomical coverage. Approximately 70% of failures were covered inside RTOG volumes, which indicates that if RTOG volume-directed planning had been used for radiation treatment in such high-risk patients, these geographical misses could have been avoided. There is also a possibility that patients with high-risk features will recur more, even after adequate coverage of target nodal regions, and it may indicate that tumor biology can also play a role in such recurrences. This study also suggests that adopting RTOG guidelines for volume delineation in high-risk cases with aggressive histology might be beneficial. However, further follow-up and meticulous documentation of the recurrences are needed to improve their understanding.
